# Unrepaired Congenital Heart Disease in Childhood: A Case of Ventricular Septal Defect and Double-Chambered Right Ventricle

**DOI:** 10.7759/cureus.78435

**Published:** 2025-02-03

**Authors:** Andres S Zamora, Antonio Escorcia, Byron R Larios Alemán, Enamaria Villalta Garcia, Christopher K Romero Ríos, Erick Gutierrez, Gery Castrillo Borge

**Affiliations:** 1 Radiology, Hospital Militar Escuela "Dr. Alejandro Dávila Bolaños", Managua, NIC; 2 Cardiology, Hospital Militar Escuela "Dr. Alejandro Dávila Bolaños", Managua, NIC; 3 Medicine, Hospital Militar Escuela "Dr. Alejandro Dávila Bolaños", Managua, NIC; 4 Medicine, Universidad Americana (UAM), Managua, NIC; 5 Internal Medicine, Hospital Militar Escuela "Dr. Alejandro Dávila Bolaños", Managua, NIC

**Keywords:** congenital heart disease (chd), double-chambered right ventricle, right ventricular outflow tract obstruction, subinfundibular hypertrophy, ventricular septal defect

## Abstract

Congenital heart diseases are structural abnormalities of the heart with functional repercussions on the patient's hemodynamics. At the time of diagnosis, most patients are in childhood, and the clinical course exhibits a wide spectrum of severity, ranging from mild conditions that do not affect the quality of life to severe presentations that may lead to death. Under this concept, we present the case of a 45-year-old patient who was diagnosed in childhood with a ventricular septal defect for which he did not receive any treatment or intervention. Despite this, he remained asymptomatic until his fourth decade, when he developed moderate exertional dyspnea associated with chest pain relieved by rest. On physical examination, a holosystolic murmur radiating to all auscultatory areas throughout the cardiac cycle was observed. Diagnostic tests such as echocardiography confirmed the presence of a pseudomembranous ventricular septal defect associated with left ventricular remodeling and hypokinesia. A chest X-ray revealed a normal cardiothoracic ratio, and a computed tomography angiogram evidenced an obstruction in the right ventricular outflow tract attributed to subinfundibular hypertrophy. Cardiac catheterization ruled out ischemic etiology. These findings led the cardiothoracic surgical team to recommend surgical intervention, which consisted of resection of the fibromuscular ridge in the right ventricular outflow tract and repair of the ventricular septal defect. Post-surgical follow-up echocardiography revealed normal hemodynamic values. This case highlights the importance of the early detection and treatment of congenital heart diseases as well as the significance of a comprehensive diagnostic approach for each patient.

## Introduction

Congenital heart diseases arise from the abnormal development of the heart, affecting up to 9.4 per 1,000 live births [1]. Diagnosis requires a comprehensive evaluation of the child and the use of complementary diagnostic tools such as echocardiography, computed tomography (CT), and magnetic resonance imaging (MRI), among others. The prognosis of these conditions varies depending on the type and severity of the defect; however, survival into adulthood has significantly improved, reaching up to 90% in Western countries [2]. Among these anomalies are ventricular septal defects (VSDs), which coexist with another structural abnormality known as double-chambered right ventricle (DCRV) in up to 44.2% of cases [3]. This association may result from a shared pathophysiology, as the turbulent flow caused by the septal defect promotes a tissue transition from endothelial to mesenchymal cells in the right ventricle. This process, driven by shear stress and mechanical strain, triggers fibroblast activation and extracellular matrix deposition, ultimately forming a band of fibroelastic tissue that progressively obstructs the right ventricular outflow tract and functionally divides the chamber in two [4].

This case report describes the coexistence of both conditions in a 45-year-old patient who was diagnosed in childhood with a VSD that was not repaired and presented with clinical manifestations in his fourth decade, leading to a new diagnostic approach that identified a DCRV.

## Case presentation

A 45-year-old male patient, with a BMI of 32.1 and no family history or toxic habits, presented to the emergency department with moderate exertional dyspnea of two months' duration, associated with chest pain (6/10 intensity) without radiation. The pain was relieved by rest, while exertion exacerbated it. Physical examination revealed an apical heartbeat visible in the left fifth intercostal space at the midclavicular line, without parasternal lift. No physical signs of a pulsatile wave were noted in the second or third left intercostal spaces. On auscultation, a holosystolic murmur was heard, radiating like a "bandolier" to all auscultatory areas. It is noteworthy that the patient had been diagnosed with congenital heart disease, a perimembranous interventricular communication, during childhood. He was previously considered for surgery between the ages of two and eight years, but his mother declined the procedure.

**Figure 1:**
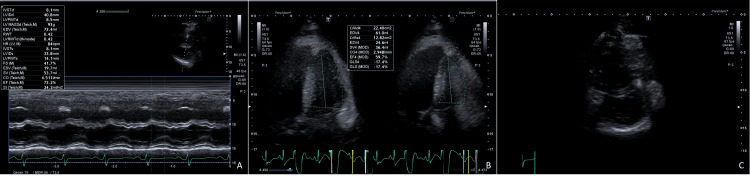
Echocardiographic ventricular function and interdependence. (A) Preserved shortening fraction, concentric remodeling of the left ventricle, and diastolic displacement of the septal wall to the left. (B) Ejection fraction by the biplane disc method (Simpson) in four chambers normal (59%). (C) Ventricular interdependence with diastolic displacement of the septum to the left (volume overload).

**Figure 2:**
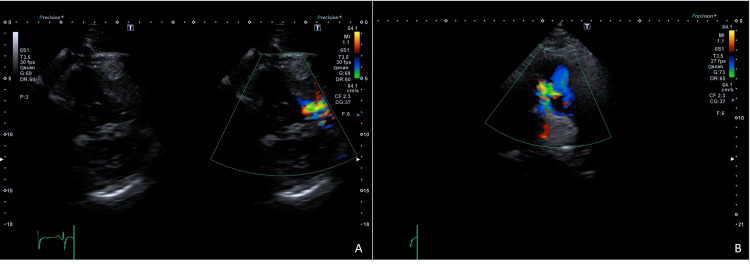
Interventricular communication by color Doppler echocardiography. (A) Parasternal short-axis view at the basal level and large vessels. (B) Systolic left-to-right flow jet.

Laboratory tests were performed, showing polycythemia (Table 1). An echocardiogram revealed the concentric remodeling of the left ventricle with hypokinesia and preserved left ventricular ejection fraction (LVEF), along with mild right ventricular dilation. A "D-sign" was noted in diastole, suggesting volume overload (Figure 1). Additionally, a perimembranous VSD with left-to-right shunting was identified (Figure 2). The valves demonstrated normal function, with gradients and velocities within normal ranges.

**Table 1:** Laboratory exams. MCV: mean corpuscular volume; MCH: mean corpuscular hemoglobin; PTT: partial thromboplastin time; PT: prothrombin time; INR: international normalized ratio

Laboratory test	Results	Reference range
White blood cell (count)	6.83 10³/µL	4.0-11.0 10³/µL
Hematocrit	52%	39-50%
Hemoglobin	18.2 g/dL	13-17 g/dL
Red blood cell (count)	5.96 10⁶/µL	4-6.3 10⁶/µL
MCV	87.2 fL	72-96 fL
MCH	30.5 pg	27-32 pg
Platelets	198 10³/µL	150-500 10³/µL
Creatinine	1.06 mg/dL	0.7-1.2 mg/dL
Glucose	99.2 mg/dL	82-115 mg/dL
PTT	32.7 sec	22.7-32.5 sec
PT	12.3 sec	9.6-12 sec
INR	1.21	0.8-1.2

**Figure 3:**
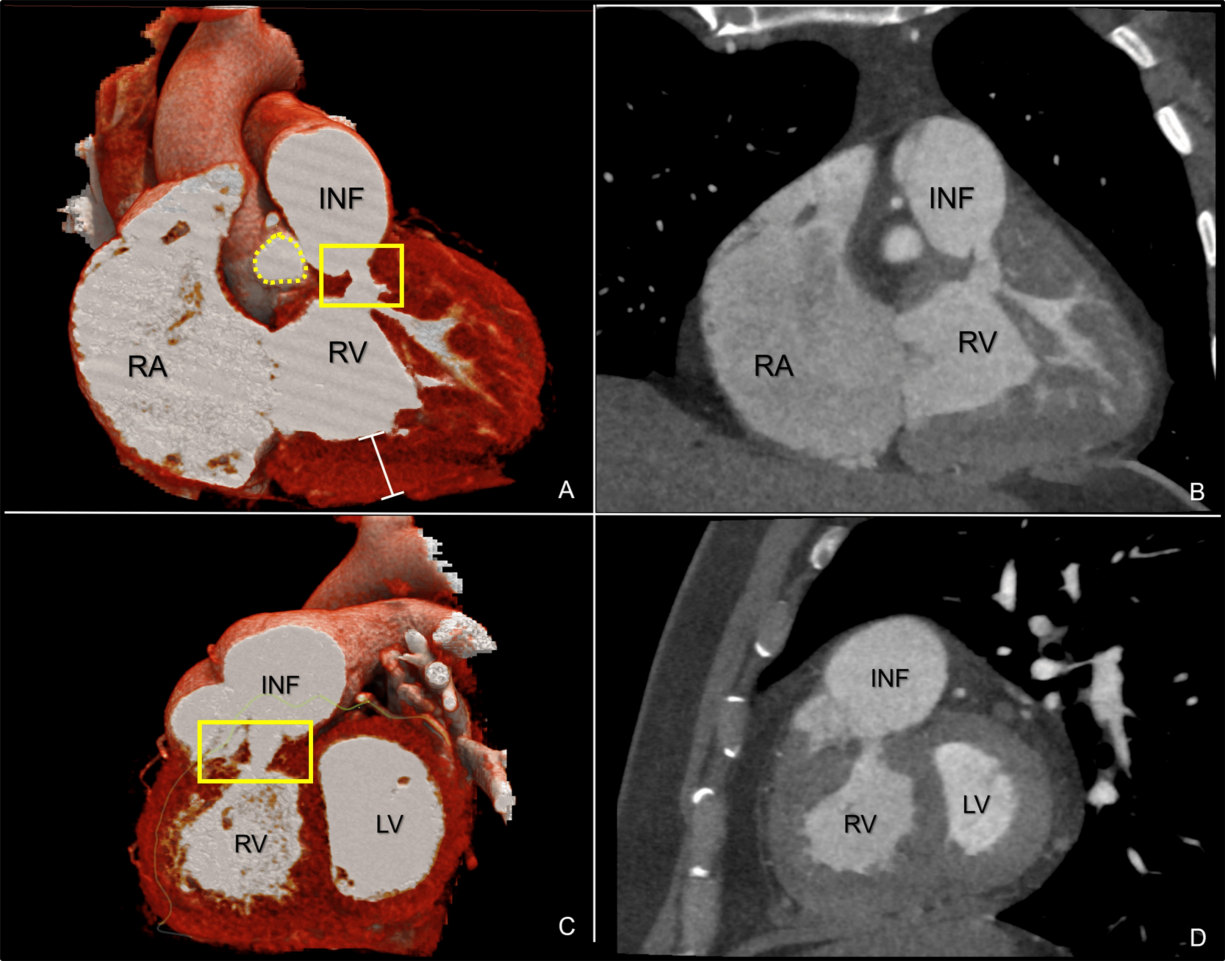
(A, B) Cardiac angiotomography, coronal cut at 52 ms phase and 3D reconstruction. (C, D) Cardiac angiotomography, sagittal cut at 52 ms phase and 3D reconstruction. The RV is shown with subinfundibular hypertrophy, indicated by white lines. A significant hypertrophy is observed at the subinfundibular level. Additionally, in the yellow box, the area of stenosis secondary to hypertrophy and the muscular band is visible. The dotted yellow circle marks the anterior pulmonary valve. RA: right atrium; RV: right ventricle; INF: infundibulum; yellow square: stenosis secondary to hypertrophy and muscular band; white line: myocardial hypertrophy

**Figure 4:**
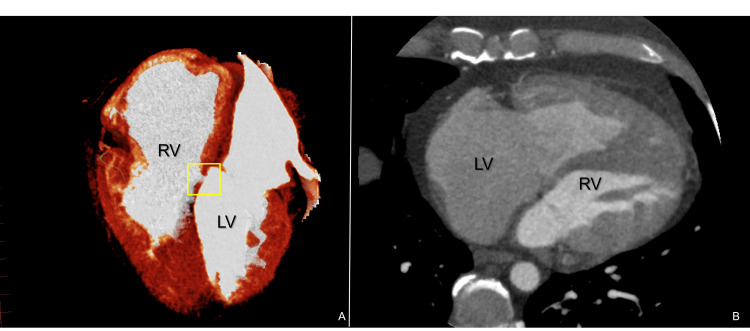
(A, B) Cardiac angiotomography axial slice and 3D reconstruction. The RV and LV are shown, with a 4×3 mm interatrial communication. RV: right ventricle; LV: left ventricle; yellow square: perimembranous interventricular communication

A chest radiograph (PA) was sent, and a cardiothoracic index of 0.54 was determined. In order to investigate the patient's condition, a CT coronary angiogram was performed, revealing an obstruction in the right ventricular outflow tract, attributed to subinfundibular hypertrophy (Figure 3) and a perimembranous interventricular communication (Figure 4). Additionally, a coronary catheterization was performed, ruling out significant lesions in the coronary tree, thus suggesting a non-ischemic etiology.

In collaboration with the cardiothoracic surgery department, a surgical procedure was decided upon, supported by the patient's symptomatic condition. During the surgery, a fibromuscular ridge was observed below the pulmonary valve, extending throughout the infundibulum. This finding was attributed to the obstruction in the right ventricular outflow tract. As a result, resection of the ridge was performed to enlarge the right ventricular outflow tract, and the interventricular communication was closed using a bovine pericardial patch.

A transesophageal echocardiogram was performed immediately after the surgery, which showed a wide right ventricular outflow tract, with a maximum residual gradient of 9 mmHg and a mean gradient of 5 mmHg, without evidence of residual shunts. The systolic excursion of the tricuspid annulus plane was 14 mm, and the left ventricular ejection fraction was 60%. The patient was subsequently transferred to the postoperative unit for complex surgeries (UPOCC). In terms of hemodynamic management, milrinone and norepinephrine were initially used and gradually withdrawn during the first 24 hours. During this period, the patient maintained adequate perfusion pressures and did not experience any arrhythmias.

At admission, polycythemia was documented; however, during the postoperative period, hemoglobin levels dropped significantly, reaching 50% of the baseline value. A blood transfusion was performed without complications, achieving a hemoglobin level of 12.1 g/dL, while other laboratory parameters remained within normal limits. On the fourth postoperative day, the patient was transferred to the cardiology ward, where he showed satisfactory progress under treatment with sildenafil, a phosphodiesterase-5 inhibitor used to reduce pulmonary vascular resistance and facilitate postoperative adaptation. Given his favorable evolution and the absence of complications, he was discharged on the seventh day with instructions for a follow-up cardiology consultation in one month and a scheduled echocardiogram in two months.

## Discussion

VSDs are one of the most common congenital heart anomalies. The incidence of VSD varies across studies and populations, but it is generally found to be between 1.5 and 3.5 per 1,000 live births [5]. VSDs are primarily diagnosed through echocardiography; this method allows for the characterization of the type, size, and number of defects, as well as the assessment of hemodynamic repercussions in detail using Doppler technology, mainly referencing pulmonary gradients, in order to make a therapeutic decision, whether surgical or interventional [6].

Regarding the natural progression of VSDs, many close spontaneously during the first year of life. Perimembranous defects are the most common, representing approximately 68.8% of cases in some studies. These defects have a lower rate of spontaneous closure compared to muscular defects, with reported closure rates ranging between 12.5% and 51.4% [7]. Those that do not close progress with pulmonary hyperflow, with the main consequence being pulmonary hypertension, leading to an increased venous return to the left chambers, which may induce the remodeling of these chambers as a compensatory mechanism, though this depends on the severity of the defect. Additionally, pulmonary hypertension induces the remodeling of the right chambers due to increased afterload. Together, these defects have significant hemodynamic repercussions for these patients [8,9]. Therefore, the persistence of this defect in the reported patient may have been one of the mechanisms that led to functional deterioration.

Additionally, the patient was identified as having subvalvular pulmonary stenosis. This defect includes infundibular pulmonary stenosis and DCRV. Infundibular stenosis can be primarily caused by discrete fibromuscular obstruction or hypertrophic cardiomyopathy. Discrete subpulmonary fibromuscular obstruction is described as a fibromuscular ring or diaphragm located in the infundibular ostium or within the infundibulum of the right ventricle. This obstruction can lead to the progressive narrowing of the right ventricular outflow tract, causing an obstruction in the pulmonary outflow [10]. In turn, this change in right ventricle architecture leads to the formation of DCRV, which presents with the division of the right ventricular outflow tract by hypertrophied muscle bundles and fibrous tissue, resulting in the formation of two distinct chambers within the right ventricle: a proximal high-pressure chamber (right ventricular sinus) and a distal low-pressure chamber (infundibular chamber) [11].

The coexistence of these findings is to be expected, as DCRV is often associated with congenital anomalies, with VSD, particularly central perimembranous VSD, being the most common [12]. In a way, the VSD precipitates the septation of the right ventricular outflow tract for the formation of the double chamber in the right ventricle, as the turbulent flow caused by the interventricular communication is responsible for a mesenchymal transition (EndMT) in this tract, leading to the formation of fibroelastic tissue that divides the ventricle. The hemodynamic impact of each of these defects is interconnected, as the VSD leads to increased pulmonary flow, thus favoring pulmonary hypertension, which, due to increased afterload, induces the constant worsening of the anatomical remodeling of this chamber [13,14]. All of this leads to patients exhibiting symptoms similar to those in this case, such as effort-induced dyspnea and chest pain.

Diagnosing DCRV can be challenging, as traditional imaging methods, such as transthoracic and transesophageal echocardiography, do not always provide definitive results, especially when there is coexistence of two malformations, as in this case [15,16]. CT is another option that is currently recognized for its high sensitivity (89.7%) and specificity (78.3%) for these anomalies, as evidenced in this case, where it was the method that allowed for the diagnosis to be made, surpassing echocardiography [17]. Advanced imaging techniques, such as cardiovascular magnetic resonance (CMR) and cardiac catheterization, are often necessary to confirm the diagnosis and assess the hemodynamic impact of the condition [15,16]. DCRV typically presents in childhood or adolescence but can also be diagnosed in adulthood, especially in patients with associated congenital anomalies like VSD, as happened with this patient, whose condition progressed to cause increasingly disabling symptoms. Approximately 3-7% of patients with VSD may develop an obstruction in the pulmonary outflow tract, highlighting the importance of early detection and intervention in these cases, considering that those with these malformations tend to evolve toward more severe hemodynamic repercussions [17].

The management of DCRV generally involves the surgical resection of the muscle bundles dividing the right ventricle, thereby relieving the obstruction of the right ventricular outflow tract. At the same time, the VSD is closed, fully correcting the genesis of the physiological and hemodynamic changes, as was done in this patient [9]. This procedure aims to restore normal flow dynamics and reduce symptoms, such as exertional dyspnea, syncope, and signs of right heart failure. Surgical outcomes are usually favorable, and recurrence is infrequent, as is the need for reintervention for residual obstructions; this is reflected in the fact that most patients experience significant improvement in symptoms and functional status after the procedure [18].

## Conclusions

This case highlights the importance of an integrated diagnostic and therapeutic approach in adult patients with untreated congenital heart defects, specifically a perimembranous VSD that, combined with an infundibular pulmonary stenosis and a DCRV, caused progressive obstruction in the right ventricular outflow tract. The patient's clinical presentation, characterized by progressive dyspnea and chest pain, was not initially clear on the echocardiogram, as it did not show conclusive findings. However, the CT angiogram emerged as a critical diagnostic tool, providing a detailed evaluation of structural abnormalities and precisely guiding the surgical treatment.

The surgical management, which involved resecting the fibromuscular ridge and closing the septal defect with a bovine pericardial patch, resulted in the resolution of the outflow tract obstruction and the elimination of residual shunts. Post-surgical results were successful, restoring cardiac function and preventing long-term complications such as pulmonary hypertension and ventricular remodeling. This case underscores the importance of the early detection and timely treatment of congenital heart defects to avoid functional deterioration. Additionally, it highlights the significance of advanced imaging techniques, such as CT angiography, in optimizing surgical outcomes, particularly in resource-limited settings, improving the prognosis for patients with complex heart conditions.
